# Risk factors associated with mechanical ventilation, autonomic nervous dysfunction and physical outcome in Vietnamese adults with tetanus

**DOI:** 10.1186/s41182-021-00336-w

**Published:** 2021-06-21

**Authors:** Rachel Davies-Foote, Truong Ngoc Trung, Nguyen Van Thanh Duoc, Du Hong Duc, Phung Tran Huy Nhat, Vo Thi Nhu Trang, Nguyen Thi Kim Anh, Pham Thi Lieu, Duong Bich Thuy, Nguyen Thanh Phong, Nguyen Thanh Truong, Pham Ba Thanh, Dong Thi Hoai Tam, Tran Thi Diem Thuy, Pham Thi Tuyen, Thanh Tran Tan, James Campbell, Le Van Tan, Zudin Puthucheary, Lam Minh Yen, Nguyen Van Hao, C. Louise Thwaites

**Affiliations:** 1 London School of Hygiene and Tropical Medicine, London, UK; 2 Hospital for Tropical Diseases, Ho Chi Minh City, Vietnam; 3 Oxford University Clinical Research Unit, Ho Chi Minh City, Vietnam; 4 Kings College, London, UK; 5 Gia Dinh Hospital, Da Nang City, Vietnam; 6 University of Medicine and Pharmacy, Ho Chi Minh City, Vietnam; 7 Centre for Tropical Medicine and Global Health, University of Oxford, Oxford, UK; 8 William Harvey Research Institute, Barts and The London School of Medicine and Dentistry, Queen Mary University of London, London, UK; 9 Royal London Hospital, London, UK

**Keywords:** Tetanus, Clostridium tetani, Tetanus toxin, Acute critical illness, Vietnam, Low-income and middle-income countries (LMICs), Severity, Long-term outcome, Physical function

## Abstract

**Background:**

Tetanus remains common in many low- and middle-income countries, but as critical care services improve, mortality from tetanus is improving. Nevertheless, patients develop severe syndromes associated with autonomic nervous system disturbance (ANSD) and the requirement for mechanical ventilation (MV). Understanding factors associated with worse outcome in such settings is important to direct interventions. In this study, we investigate risk factors for disease severity and long-term physical outcome in adults with tetanus admitted to a Vietnamese intensive care unit.

**Methods:**

Clinical and demographic variables were collected prospectively from 180 adults with tetanus. Physical function component scores (PCS), calculated from Short Form Health Survey (SF-36), were assessed in 79 patients at hospital discharge, 3 and 6 months post discharge.

**Results:**

Age, temperature, heart rate, lower peripheral oxygen saturation (SpO_2_) and shorter time from first symptom to admission were associated with MV (OR 1.03 [ 95% confidence interval (CI) 1.00, 1.05], *p* = 0.04; OR 2.10 [95% CI 1.03, 4.60], *p* = 0.04; OR 1.04 [ 95% CI 1.01, 1.07], *p* = 0.02); OR 0.80 [95% CI 0.66, 0.94], *p* = 0.02 and OR 0.65 [95% CI 0.52, 0.79, *p* < 0.001, respectively).

Heart rate, SpO_2_ and time from first symptom to admission were associated with ANSD (OR 1.03 [95% CI 1.01, 1.06], *p* < 0.01; OR 0.95 [95% CI 0.9, 1.00], *p* = 0.04 and OR 0.64 [95% CI 0.48, 0.80], *p* < 0.01, respectively).

Median [interquartile range] PCS at hospital discharge, 3 and 6 months were 32.37 [24.95–41.57, 53.0 [41.6–56.3] and 54.8 [51.6–57.3], respectively. Age, female sex, admission systolic blood pressure, admission SpO_2_, MV, ANSD, midazolam requirement, hospital-acquired infection, pressure ulcer and duration of ICU and hospital stay were associated with reduced 0.25 quantile PCS at 6 months after hospital discharge.

**Conclusions:**

MV and ANSD may be suitable endpoints for future research. Risk factors for reduced physical function at 3 months and 6 months post discharge suggest that modifiable features during hospital management are important determinants of long-term outcome.

**Supplementary Information:**

The online version contains supplementary material available at 10.1186/s41182-021-00336-w.

## Background

Tetanus is a vaccine-preventable disease associated with severe muscle spasm and cardiovascular system disturbance that remains a common cause of acute critical illness in low- and middle-income countries (LMICs) [1].

As critical care services improve throughout the world, the capacity to manage severe tetanus is increasing and some centres have reported significant reductions in mortality rates [2–4]. Nevertheless, patients still experience severe muscle spasms necessitating prolonged treatment with muscle relaxants and mechanical ventilation in addition to cardiovascular instability and autonomic nervous system dysfunction (ANSD) [5].

Historical literature contains many studies documenting risk factors for mortality in tetanus when muscle spasm were the principal causes of death [6, 7]. It is unclear whether these same risk factors are important in modern ICU settings where death due to respiratory muscle spasms can be prevented by mechanical ventilation [5, 8]. Furthermore, as mortality rates decrease, predicting those at risk of other outcomes such as mechanical ventilation or ANSD may be more relevant to everyday clinical practice and clinical trial outcomes [9, 10].

As survivorship improves, understanding the quality of life of survivors is increasingly important but is much neglected in published literature, particularly in LMIC settings. There is very little known about disability in tetanus survivors although this is particularly important as in most countries tetanus affects mainly men of working age where long-term disability might have a significant social and economic impact. The limited data that do exist suggest a significant number of survivors are left with impairment. In a retrospective study of adults with tetanus admitted to intensive care units (ICUs) in France, out of 38 survivors with a known functional status at follow-up, 39% suffered worsening of functional impairment compared to before admission, whilst 17% required admission to a long-term care facility [11]. In a Japanese case series, 35% of all survivors were discharged from hospital to a facility other than home [12]. Muscle wasting has been identified as an important factor contributing to ICU-related functional impairment [13]. We have previously reported significant muscle wasting during hospitalisation in adults with tetanus, the degree of which was related to age, occurrence of hospital-acquired infection, and subsequent health-related quality of life scores [14].

We examine what factors predict more severe disease and also what factors are associated with physical disability at hospital discharge, 3 and 6 months post hospital discharge. This information is an important step to improving tetanus outcome in other centres and understanding more about long-term effects of critical illness in low- and middle-income countries.

## Methods

The study was performed at the Hospital for Tropical Diseases (HTD), Ho Chi Minh City. The hospital is a tertiary referral centre for infectious diseases serving Southern Vietnam. Patients were treated according to a standard protocol as previously described [15]. Briefly, this consists of antibiotics and antitoxin; spasm control with benzodiazepeines (midazolam or diazepam) escalated to non-depolarising muscle relaxants (pipecuronium) and mechanical ventilation. Airway management is by primary tracheostomy with the following indications: laryngeal spasms compromising the airway, sputum retention and to facilitate mechanical ventilation. ANSD is managed with magnesium sulphate and additional calcium antagonists or inotropes as clinically indicated. All patients receive a full primary course of tetanus-toxoid containing vaccine initiated at discharge. ANSD was defined as at least 3 of (i) heart rate > 100 beats per minute (bpm), (ii) systolic blood pressure > 140 mmHg, (iii) mean arterial pressure < 60 mmHg, (iv) pyrexia > 38 °C, and (v) fluctuating blood pressure, occurring within one day with no other apparent cause [16].

Investigation of risk factors for tetanus severity was carried out with prospectively collected data from two cohorts of patients ≥ 16 years old admitted to the hospital’s ICU with a diagnosis of generalised tetanus: from August 2016 to March 2017 and January to July 2018, described elsewhere [14]. Physical functional outcome was evaluated in the subgroup of patients enrolled between August 2016 and March 2017 in whom there were 2 exclusion criteria: not speaking Vietnamese and unable to walk before admission. The Study Flowchart is shown in Additional File 1.

Data were collected prospectively daily by study staff in all enrolled subjects. A core dataset of recognised tetanus prognostic features, basic clinical and demographic data were collected in both cohorts Additionally patients were followed daily for complications and interventions such as MV by study staff. Treatment for hospital-acquired infections of ventilator-associated pneumonia, urinary tract infection and bacteraemia were specifically evaluated in these daily observations. Diagnosis and treatment of these followed local hospital guidelines. In the physical outcome subgroup, physical function was assessed using the physical component summary score (PCS) derived from the RAND 36-item Short Form Health Survey (SF-36) version 1 completed at hospital discharge by face-to-face interview, 3 months post discharge and 6 months post discharge by telephone interview [17]. Using the method described by Ware et al., individual question scores were transformed and averaged to the eight aggregate scale (domain) scores and, from these, the PCS was calculated [18]. This method results in a single PCS for each patient, calculated by differentially weighting then combining the scale scores and applying a linear transformation. Applying this methodology to the US population data results in a mean PCS of 50 with a standard deviation of 10. Standard US scores were used for PCS calculation as no validated Vietnamese population standard scores exist.

### Statistical analysis

Descriptive statistics were used to describe the sample with the median and interquartile range (IQR) for continuous data, and count and percentage for categorical data. Univariate logistic regression was used to examine associations between ANSD and each of the demographic and baseline clinical features. Features were selected based on the availability in the core dataset (i.e. both cohorts of patients) and clinical plausibility associated with known prognostic indicators in tetanus. Baseline features selected were age, gender, entry site, incubation period, period of onset, time from first symptom to first spasm and symptom of difficulty breathing on admission. Clinical features on admission were taken as the worst value in the first 24 h: highest heart rate, heart rate range, maximum systolic blood pressure and temperature, peripheral oxygen saturation (SpO_2_), serum creatinine, platelet count, white blood cell count, urine output and Ablett score. Briefly, Ablett scores the severity of tetanus according to spasms (grade 1 no spasms, 2 mild spasms, 3 spasms interfering with respiration) and additional clinical autonomic disturbance (grade 4) [19]. Variables that were associated with ANSD in the univariate analysis were entered into a multivariate logistic regression model using a backward approach dropping one least significant variable at a time. A backward approach was selected due to the large number of potential covariates and to reduce issues related to multiple collinearity. The major assumptions of logistic regression including the linearity assumption, multicollinearity and influential values were checked for the models.

The same strategy as outlined above was used to evaluate risk factors for MV but given the independent variables being used to predict the requirement for MV included baseline clinical features recorded during the first 24 h of ICU admission, those patients who were also mechanically ventilated during the first 24 h of admission were excluded from this univariate and multivariate logistic regression analysis.

Prior to analysis by linear regression, the PCS at hospital discharge was graphed as a histogram and quantile-quantile (Q-Q) plot to assess the distribution of residuals and validate suitability for linear regression analysis. Simple linear regression was used to examine the dependency of PCS at hospital discharge by each of the independent variables in the dataset. Multiple linear regression was then used to model variables associated with PCS at hospital discharge. Variables that were identified to be significantly associated with PCS at hospital discharge in analysis by simple linear regression were all entered into the model using a backwards approach, dropping one least significant variable at a time. The model was then verified using a forward approach by introducing the variables sequentially. The distribution of the PCS at 3 months and 6 months post discharge were very skewed (Shapiro-Wilks normality tests *p* values < 0.000001 for 3 month and 6 month PCS) with mode values at the extreme of the range. Linear quantile was therefore used for analysis as this, unlike parametric methods, makes no assumptions about the distribution of the residuals. Using this method, the effect of independent variables on admission to ICU and features during ICU management were modelled on each specified quantile of 0.25, 0.5 and 0.75 (i.e. 1st quartile, median and 3rd quartile) of the respective dependent variables: 3-month post discharge PCS, and 6-month post discharge PCS. The threshold for significance was *p* ≤ 0.05 (two-tailed). All analyses were carried out in Stata (StataCorp) version 16 and R Version 4.0.2 (R Corporation, Vienna).

This study was approved by the London School of Hygiene and Tropical Medicine (LSHTM) ethics committee, the Oxford Tropical Medicine Ethics Committee (OxTREC) and the local HTD ethics committee. All participants gave written informed consent prior to enrolment.

## Results

One hundred eighty patients aged between 17 and 98 years, with generalised tetanus, were included in this study. A summary of the baseline features of the patients are described in Table 1. Ninety of 180 (50%) required MV for a median duration of 16.0 [IQR 12–24] days (Additional Files 1 & 2).

**Table 1:** Description of the demographic and clinical features of tetanus patients on admission to ICU

	Physical outcome cohort (*N* = 80)	All (*n* = 180)
Parameter	Median [IQR] or count (%)	Median [IQR] or count (%)
Demographics		
Age (years)	49.0 [35.0–59.5]	51.0 [40.5–62.0]
Male sex	64 (80.0%)	143 (79.4%)
Coexisting comorbidities	22 (27.5%)	73 (40.6%)
Tetanus specific features		
Deep wound	2 (2.5%)	5 (2.8%)
Incubation period (days)	8 [6–11]	8 [7–13]
Onset period (h)	48 [24–72]	48 [24–72]
Time from first symptom to admission (days)	3 [2–5]	3 [2–5]
History of difficulty breathing	16 (20.0%)	37 (20.6%)
Clinical features during first 24 h of admission		
Highest systolic blood pressure (mmHg)	130 [120–140]	140 [130–150]
Highest heart rate (bpm)	90 [82–98]	93 [84–104]
Highest temperature (°C)	37.0 [37.0–37.5]	37.5 [37.0–38]
SpO_2_ on admission (%)	97 [96–98]	97 [95–98]
Ablett score:*		
1	17 (21.3%)	36 (20%)
2	49 (61.3%)	116 (64.4%)
3	13 (16.3%)	27 (15%)
4	1 (0.6%)	1 (0.6%)

*Ablett score: Grade 1 no spasms, grade 2 mild spasms not interfering with respiration, grade 3 spasms interfering with respiration and grade 4: grade 3 with clinical signs of autonomic nervous system dysfunction [19]

### Associations of admission features with disease severity

After removing the 16 patients who were mechanically ventilated during the first 24 h of admission (including 2 patients who died), data from 164 patients were available for analysis, of whom 74 (45.1%) patients required MV and 3 (1.8%) died. The features on ICU admission associated with MV after 24 h admission in univariate analysis are shown in Table 2.

**Table 2:** Tetanus risk factors for mechanical ventilation (*n* = 164)

Parameter	No MV (***n*** = 90)	MV (***n*** = 74)	OR for MV	95% CI	***P*** value
	Median [IQR], mean (±SD) or count (%)				
Age (years)	46 [37–59]	52.5 [43–65]	1.027	1.01–1.05	0.01
Male sex	72 (80.0%)	60 (81.1%)	1.07	0.49–2.36	0.86
Any comorbidities	30 (33.3%)	34 (45.9%)	1.70	0.90–3.22	0.10
Deep wound	2 (2.2%)	2 (2.7%)	1.22	0.14–10.39	0.84
Incubation period (days) (*n* = 122)	10 [7–14]	8 [6–11]	0.99	0.952–1.04	0.78
Period of onset (h) (*n* = 141)	48 [24–72]	48 [24–48]	0.98	0.97–0.99	0.004
Time from first symptom to admission (days) (*n* = 162)	4 [3–6]	3 [2–4]	0.74	0.62–0.86	< 0.001
History of difficulty breathing	10 (11.1%)	19 (25.7%)	2.76	1.22–6.62	0.02
Clinical Features during first 24 h of admission					
Highest SBP (mmHg)	136 [120–150]	140 [130–147.5]	1.01	0.99–1.03	0.38
Highest heart rate (bpm)	90 [82–99.5]	97 [88–111.5]	1.05	1.03–1.08	< 0.001
Heart rate range (bpm)	12 [8–20]	16 [10–26]	1.05	1.02–1.08	0.001
Highest temperature (°C)	37.4 [37.0–37.6]	37.5 [37.0––38.0]	2.35	1.36–4.28	0.003
SpO_2_ (%)	97 [96–98]	96 [94–97]	0.82	0.700–0.93	0.01
SOFA* score = 0	83 (92.2%)	52 (70.3%)	Ref.		
SOFA* score > = 1	7 (7.8%)	22 (29.7%)	5.02	2.09–13.45	0.001
Urine output (ml/h)	69.7 [42.2–125.5]	76.6 [42.9–113.3]	1.00	0.10–1.00	0.81
Creatinine (μmol/L)	78.9 (± 16.4)	90.9 (± 48.4)	1.01	1.00–1.03	0.04
Platelets (× 10^9^/L)	283.6 (± 81.7)	282.8 (± 97.4)	1.00	1.00–1.00	0.96
Potassium (mEq/L) (*n* = 94)	3.6 (± 0.4)	3.7 (± 0.6)	1.59	0.72–3.75	0.27
White blood count (× 10^9^/L) (*n* = 95)	9.6 [7.1–11.0]	10.2 [8.4–13.0]	1.15	1.02–1.34	0.04

*Sequential organ failure score [20]. Descriptive statistic describe number of patients with SOFA score = 0 (reference) or > = 1

Multivariate logistic regression analysis showed that the following admission variables were associated with increased odds of requiring MV: increased age (OR 1.03, 95% CI 1.00–1.05, *p* = 0.04), temperature (OR 2.10, 95% CI 1.02–4.58, *p* = 0.04) and HR (OR 1.04, 95% CI 1.01–1.07, *p* = 0.02); and lower SpO_2_ (OR 0.80, 95% CI 0.66–0.94, *p* = 0.02) and shorter time from first symptom to admission (OR 0.65, 95% CI 0.52–0.79, *p* < 0.001).

Analysis of features present at ICU admission and associated with the development of the ANSD was performed on data from 177 participants after removing 1 patient with ANSD on admission to ICU and 2 patients with missing data. In this group, 43 (24.3%) developed ANSD. The features present at ICU admission and associated with the development of ANSD in univariate analysis after excluding the one patient who had ANSD on admission are shown in Table 3.

**Table 3:** Tetanus risk factors for development of ANSD (*n* = 179)

Parameter	No ANSD (***n*** = 135)	ANSD (***n*** = 44)	OR for ANSD	95% CI	***P*** value
	Median [IQR], mean (± SD) or count (%)				
Age (years)	49 [40–61]	52.5 [45–68.5]	1.02	0.10–1.04	0.08
Male sex	108 (80%)	35 (79.5%)	0.97	0.429–2.37	0.95
Any comorbidities	51 (37.8%)	21 (47.7%)	1.50	0.75–3.00	0.24
Deep wound	3 (2.2%)	1 (2.3%)	1.02	0.05–8.23	0.98
Incubation period (days) (*n* = 134)	10 [7–14]	7 [5–10]	0.10	0.95–1.04	0.83
Period of onset (h) (*n* = 156)	48 [24–72]	24 [24–48]	0.98	0.96–0.99	0.004
Time from first symptom to admission (days) (*n* = 177)	4 [3–5]	2 [2–3]	0.69	0.54–0.85	0.001
History of difficulty breathing	23 (17.0%)	14 (31.8%)	2.27	1.03–4.92	0.04
Clinical Features during first 24 h of admission					
Highest SBP (mmHg)	140 [125–150]	140 [130–150]	1.02	1.00–1.04	0.02
Highest heart rate (bpm)	92 [84–100]	96 [88–120]	1.03	1.01–1.05	0.002
Heart rate range (bpm)	14 [8–22]	16 [10–28]	1.02	0.99–1.05	0.06
Highest temperature (°C)	37.4 [37–37.8]	37.5 [37.2–38.0]	1.46	0.850–2.49	0.16
SpO_2_ (%)	97 [96–98]	96 [93–98]	0.96	0.90–1.00	0.08
SOFA score:					
0	115 (85.2%)	31 (70.5%)	Ref.		
> = 1	20 (14.8%)	13 (29.5%)	2.41	1.06–5.36	0.03
Urine output (ml/h)	71 [43–123]	77.5 [42–110]	1.00	0.99–1.00	0.62
Ablett on admission:					
1	34 (25.2%)	2 (4.5%)	Ref.		
2	83 (61.5%)	33 (75.0%)	6.76	1.90–43.12	0.011
3	18 (13.3%)	9 (20.5%)	8.50	1.94–59.82	0.010
Creatinine (μmol/L)	82.6 (± 32.3)	96.5 (± 58.9)	1.01	1.00–1.02	0.08
Platelets (× 10^9^/L)	281.4 (± 85.1)	289.5 (± 99.6)	1.01	1.00–1.00	0.60
Potassium (mEq/L) (*n* = 99)	3.6 (± 0.5)	3.7 (± 0.6)	1.493	0.64–3.53	0.35
White blood count (× 10^9^/L) (*n* = 100)	9.7 [7.1–11.3]	10.9 [8.6–14.2]	1.167	1.04–1.34	0.02

Multivariate analysis showed similar admission features were associated with the development of ANSD as with MV. There was strong evidence of an association between the development of ANSD with time from the first symptom to admission (OR 0.64, 95% CI 0.48–0.80, *p* < 0.01) and maximum HR during the first day of admission (OR 1.03, 95% CI 1.01–1.06, *p* = 0.004). There was some evidence of an association between SpO2 on admission to ICU and development of ANSD (OR 0.95, 95% CI 0.90–0.10, *p* = 0.04).

### Physical functioning at discharge

Eighty patients with generalised tetanus admitted to the ICU at HTD between August 2016 and June 2017 had a physical function at discharge data available and were included in the subgroup analysis. The median [IQR] PCS at hospital discharge was 32.37 [24.95–41.57].

The association of independent admission features with PCS at hospital discharge are shown in Additional File 3. Independent admission features associated with PCS at hospital discharge include age, female sex, presence of comorbidities, platelet count and HR during the first 24 h of admission. The association of independent features during ICU admission with PCS at hospital discharge are shown in Additional File 4. Independent variables significantly associated with PCS at hospital discharge included the requirement for tracheostomy and duration of tracheostomy requirement, requirement for MV, development of ANSD, development of any HAI, duration of ICU admission and duration of hospital admission.

The features associated with PCS at hospital discharge in the multiple regression analysis were age (β − 0.20, 95% CI − 0.31, − 0.09, *p* < 0.001), duration of ICU admission in days (β − 0.22, 95% CI − 0.40, − 0.05, *p* = 0.01) and the development of at least one HAI (β − 4.62, 95% CI − 9.26, 0.02, *p* = 0.05).

### Physical functioning at 3 and 6 months

Median PCS at 3 months and 6 months post discharge were 53.0 [IQR 41.6–56.3] and 54.8 [IQR 51.6–57.3], respectively. Features associated with PCS in participants in the lowest functioning quantile (0.25) are shown in Tables 4 and 5. Admission features of the female sex, age, SpO_2_ and highest systolic blood pressure were significantly associated with PCS at 6 months post discharge in this quantile. Management features strongly associated with PCS at 6 months after discharge in the lowest quantile PCS included MV, development of ANSD, development of HAI, bacteraemia, ventilator-associated pneumonia, pressure ulcer, length of ICU and hospital stay (Table 5).

**Table 4:** The association between independent features on ICU admission and 25th quantile physical function at 3 months and 6 months post discharge

	3 months ***N*** = 75		6 months ***N*** = 69	
	β (95% CI)	***P*** value	β (95% CI)	***P*** value
Female sex	− 18.00 (− 26.58, − 10.33)	0.002	− 20.21 (− 23.04, − 10.13)	< 0.001
Age	− 0.46 (− 0.53, − 0.37)	< 0.001	− 0.270 (− 0.37, − 0.26)	< 0.001
Any pre-existing comorbidities	− 15.28 (− 23.85, − 7.30)	0.003	− 11.36 (− 17.81, − 1.39)	0.07
Incubation period (*n* = 50)	0.69 (− 1.137, 1.41)	0.19	0.17 (− 0.01, 0.42)	0.31
Period of onset (*n* = 55)	0.06 (− 0.13, 0.10)	0.32	0.06 (− 0.22, 0.08)	0.11
Time from 1st symptom to admission	1.19 (− 1.64, 2.10)	0.19	0.70 (− 1.17, 1.25)	0.13
Difficulty breathing on admission	− 9.61 (− 14.91, 8.08)	0.18	− 3.00 (− 16.57, 0.08)	0.35
Clinical Features during first 24 h of admission				
Highest systolic blood pressure (mmHg)*	− 0.21 (− 0.48, − 0.15)	0.07	− 0.21 (− 0.27, 0.02)	0.01
Highest heart rate (bpm)	− 0.08 (− 0.38, 0.20)	0.64	− 0.08 (− 0.40, -0.01)	0.18
Heart rate range (bpm)*	− 0.07 (− 0.42, 0.30)	0.70	− 0.13 (− 0.31, 0.01)	0.19
Highest temperature (°C)	5.07 (− 7.16, 8.76)	0.16	1.97 (− 13.28, 3.17)	0.23
SpO2 on admission (%)*	0.55 (0.40, 3.81)	0.21	0.72 (0.00, 3.26)	0.03
SOFA** score^1*^ 0 > = 1	− 6.17 (− 17.98, − 0.01)	0.27	− 3.97 (− 17.98, − 0.85)	0.82
Urine output (ml/h)	0.04 (− 0.14, 0.04)	0.20	0.01 (− 0.02, 0.03)	0.34
Creatinine (μmol/L)	0.16 (− 0.06, 0.27)	0.23	0.01 (− 0.07, 0.17)	0.93
Platelets (× 10^9^/L)	− 0.06 (− 0.10, − 0.04)	0.01	− 0.03 (− 0.08, 0.00)	0.13

^1^SOFA score of 0 is the baseline group

*During the first 24 h of hospital admission

**Sequential organ failure score [20]

**Table 5:** The association between independent features during ICU management and 25th quantile PCS at 3 months and 6 months post discharge

	3 months		6 months	
	β (95% CI)	***P*** value	β (95% CI)	***P*** value
Tracheostomy required	− 9.69 (− 17.61, − 2.65)	0.06	− 6.80 (− 17.31, − 0.73)	0.08
Duration tracheostomy (days) (*n* = 30)	− 0.47 (− 2.00, − 0.34)	0.16	− 0.72 (− 1.49 − 0.38)	0.25
Mechanical ventilation required	− 9.06 (− 17.56, 2.29)	0.10	− 11.36 (− 18.19, − 0.55)	0.02
Duration mechanical ventilation (days) (*n* = 27)	− 0.33 (− 1.37, 0.08)	0.38	− 0.36 (− 1.30, − 0.22)	0.25
Autonomic nervous system disturbance	− 13.52 (− 18.22, − 8.84)	0.02	− 15.65 (− 19.29, − 4.70)	0.001
Duration diazepam required (days) (*n* = 66)	0.51 (0.35, 0.80)	0.01	0.26 (0.10, 0.69)	0.07
Duration midazolam required (days) (*n* = 40)	− 0.39 (− 0.87, 0.21)	0.31	− 0.61 (− 1.28, 0.02)	0.02
Duration Magnesium sulphate (days) (*n* = 14)	2.06 (− 7.73, 2.79)	0.10	0.73 (− 8.58, 1.57)	0.53
Duration pipecuronium (days) (*n* = 22)	− 0.97 (− 1.15, 1.33)	0.29	− 0.69 (− 0.93, 0.90)	0.35
Ventilator-associated pneumonia*	− 1.80 (0.00, − 0.153)	0.09	− 2.24 (− 17.98, − 1.92)	0.001
Bacteraemia*	− 12.34 (− 28.53, − 1.00)	0.14	− 14.51 (− 25.34, − 2.13)	0.03
Urinary tract infection*	− 10.46 (− 15.11, 9.99)	0.21	− 11.12 (− 17.20, − 0.44)	0.12
Any healthcare associated infection	− 13.33 (− 17.92, − 3.51)	0.03	− 15.73 (− 20.04, − 4.05)	0.001
Pressure ulcer	− 12.51 (− 17.98, 7.59)	0.25	− 20.28 (− 17.98, − 14.713)	< 0.001
Length of ICU stay (days)	− 0.52 (− 0.91, − 0.29)	0.02	− 0.56 (− 0.61, − 0.32)	0.004
Length of hospital stay (days)	− 0.64 (− 0.82, − 0.30)	0.02	− 0.48 (− 0.70, − 0.34)	0.01

*Infections occurring during hospitalisation and deemed to be healthcare-related infections

In those with highest quantile PCS (0.75 quantile), ventilator-associated pneumonia was associated with reduced PCS at both 3 and 6 months after hospital discharge (β − 3.32 [95% CI − 0.34, 0.00], *p* = 0.002; β − 2.61 [95% CI − 2.61, 18.00], *p* < 0.001, respectively), and female sex at 6 months (β − 5.20 [95% CI − 5.57, − 3.21], *p* = 0.04).

## Discussion

We present data from a large contemporary series of patients with tetanus. Despite low mortality rates, our patients required long periods in ICU and developed severe tetanus with respiratory compromise and ANSD. Risk factors identified for these were similar to those previously identified for mortality [6, 21]. Whilst some variables such as SpO_2_, were novel, dyspnoea noted by admitting doctors is a recognised prognostic feature and we believe SpO_2_ represents a more objective measure of respiratory compromise [21]. The similarity between the risk factors we have identified and those identified for mortality supports their use as alternative markers of disease severity or study end-points in settings such as ours where mortality is low [22].

Our study is limited by the risk factors available for consideration, and sample size to look at smaller sub-populations. Data selection was based on published literature, including a large case series previously at our hospital. It is possible therefore that important factors were not identified or simplified markers. For example, we used a simple classification of wounds as superficial or deep based on a previous case series of 100 patients in our centre (3). Additionally, we have not included factors such as possible vaccination history. No patients reported knowledge of receiving tetanus vaccination and anti-tetanus toxoid antibodies from patients in the 2018 cohort were measured and shown to be sub-protective (reported elsewhere) but it is possible that some patients may have received some previous immunisation in infancy and had low (sub-protective) antibody levels. The Expanded Programme on Immunization was introduced into Vietnam in 1981, and since 1994 has consistently reported high coverage (90–99%) for DTP3 Similarly 89–93% for maternal vaccination [23]. It is therefore possible that particularly the younger patients may have received vaccination in infancy.

Our data on physical function at hospital discharge, 3 months post discharge and 6 months post discharge demonstrates that most of this relatively young and middle-aged population regains normal functioning after a severe illness has resolved. We identified age and prolonged hospital stay as the principal indicators of worse physical functioning at hospital discharge with HAI non-significantly associated. In those with the lowest PCS measures 6 months post hospital discharge many factors were significantly associated with reduced physical functioning. Multivariate analysis was not possible due to small numbers in our sample; however, it is likely that many of these features are indicative of severe disease or prolonged treatment in ICU. Interestingly neither duration of ventilation, tracheostomy or pipecuronium appeared to be strongly associated with reduced PCS at 3 or 6 months after discharge. This may indicate that other events, such as HAI which are also associated with prolongation of ICU and hospital stay, are more important determinants of outcome. Age and HAI were factors we have previously identified as significant contributors to increased muscle wasting during hospitalisation and were are independent of the duration of hospital length of stay [14]. We believe this study serves as further evidence that preventing muscle wasting should be a priority in improving functional outcome in critically ill patients. HAI remains a modifiable factor associated with this, and as we observed that even in those with highest physical function, ventilator associated pneumonia remained a predictor of PCS.

The only available Vietnamese SF-36 data is from a group of Vietnamese migrants to Australia which cannot be generalised to the entire Vietnamese population [24] and the lack of data regarding the use of SF-36 in the Vietnamese population is a significant limitation of our study. In lieu of robust population standard normative data, we used American population data. Additionally, whilst we used an accepted algorithm to calculate the PCS, it remains unclear whether other algorithms may be superior due to assumptions about the relationship between physical and mental health scale components—also unknown in the Vietnamese population [25]. Our results, however, are in agreement with previous individual scale analysis and by using a single component score we reduce concerns related to statistical power and multiple testing [14]. By publishing data from Vietnamese adults, we are able to contribute towards a better understanding of SF-36 in this population.

## Conclusions

We observed that risk factors for MV, development of ANSD and reduced physical functioning were consistent with existing knowledge and findings in settings of low mortality. These outcomes may therefore be suitable endpoints against which to evaluate therapeutic interventions. Risk factors for reduced functional outcome at 3 months and 6 months post discharge suggest that modifiable features during hospitalisation are important determinants of long-term outcome.

Tetanus remains, however, a vaccine-preventable disease and improved preventative strategies should continue to be central to reducing morbidity and mortality from this disease.

## Supplementary Information

**Additional file 1.** Study Flowchart.

**Additional file 2.** Flowchart of respiratory management.

**Additional file 3. **The association between independent features on ICU admission and SF 36 physical function composite score at hospital (*n* = 79).

**Additional file 4. **The association between independent features during ICU management and SF 36 physical function composite score at hospital discharge (*n* = 79).

## Data Availability

The dataset(s) supporting the conclusions of this article is(are) available in the Oxford University Research Archive (ORA).

## Abbreviations

ANSD: Autonomic nervous system dysfunction
HAI: Hospital-acquired infection
HTD: Hospital for Tropical Diseases, Ho Chi Minh City, Vietnam
ICU: Intensive care unit
IP: Incubation period
LMIC: Low- and middle-income countries
MV: Mechanical ventilation
OP: Onset period
PCS: Physical component summary score of the RAND 36-item Short Form Health Survey
SF-36: RAND 36-item Short Form Health Survey
SOFA: Sequential organ failure assessment score
SpO_2_: Peripheral capillary oxygen saturation
WHO: World Health Organization
