# Supplementary figures and images for: Risk factors associated with mechanical ventilation, autonomic nervous dysfunction and physical outcome in Vietnamese adults with tetanus
Source: Trop Med Health. 2021 Jun 21;49:50. doi: 10.1186/s41182-021-00336-w (PMC8215632; doi:10.1186/s41182-021-00336-w)

**Additional file 1: Study Flow Chart**
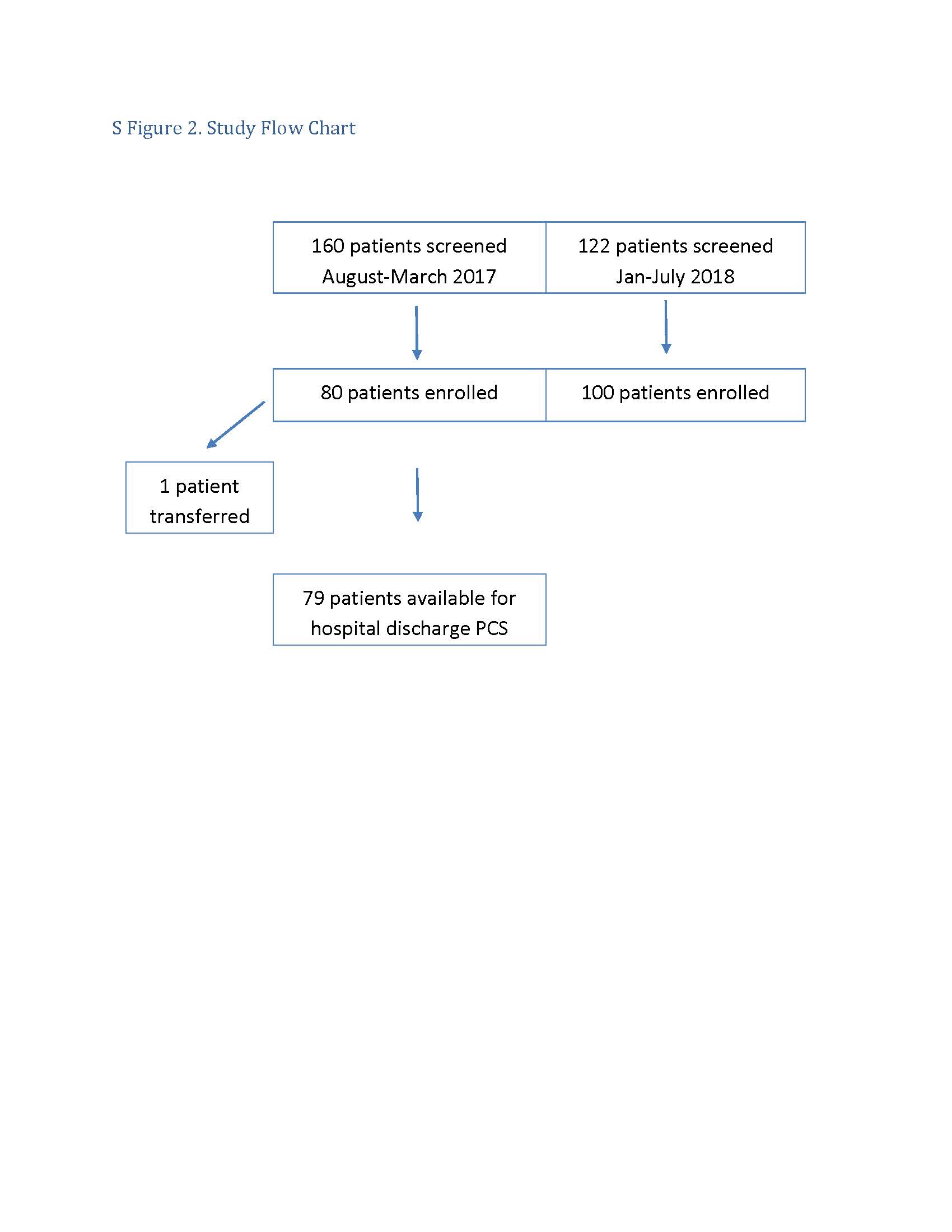

Supplement: Supplementary file 1 — Additional file 1. Study Flowchart. [file 41182_2021_336_MOESM1_ESM.docx]
