# Supplementary material for: Risk factors associated with mechanical ventilation, autonomic nervous dysfunction and physical outcome in Vietnamese adults with tetanus
Source: Trop Med Health. 2021 Jun 21;49:50. doi: 10.1186/s41182-021-00336-w (PMC8215632; doi:10.1186/s41182-021-00336-w)
Supplement: Supplementary file 2 — Additional file 2. Flowchart of respiratory management. [file 41182_2021_336_MOESM2_ESM.docx]

**Additional File 2: Flow chart of respiratory management of patients**

No pipecuronium required

(n=12)

No mechanical ventilation required

(n=4)

Tracheostomy required

(n=94)

Requirement for neuromuscular blockade with pipecuronium

(n=78)

Mechanical ventilation required

(n=90)

Enrolled adults with tetanus

(n=180)
