# Supplementary material for: Risk factors associated with mechanical ventilation, autonomic nervous dysfunction and physical outcome in Vietnamese adults with tetanus
Source: Trop Med Health. 2021 Jun 21;49:50. doi: 10.1186/s41182-021-00336-w (PMC8215632; doi:10.1186/s41182-021-00336-w)
Supplement: Supplementary file 3 — Additional file 3. The association between independent features on ICU admission and SF 36 physical function composite score at hospital (n = 79). [file 41182_2021_336_MOESM3_ESM.docx]

**Additional file 3:** The association between independent features on ICU admission and SF 36 physical function composite score at hospital discharge (n=79)

| **Parameter** | **β** | **95% CI β** | **P-value** |
| --- | --- | --- | --- |
| Female sex | -7.89 | -13.31, -2.46 | 0.01 |
| Age | -0.30 | -0.41, -0.18 | <0.001 |
| Any pre-existing comorbidities | -6.26 | -11.25, -1.26 | 0.02 |
| Incubation period (n=57) | 0.29 | -0.19, 0.78 | 0.23 |
| Period of onset (n=64) | 0.05 | -0.01, 0.10 | 0.09 |
| Time from 1^st^ symptom to admission | 0.75 | -0.14, 1.63 | 0.10 |
| Difficulty breathing on admission | -2.04 | -7.87, 3.80 | 0.49 |
| Highest systolic blood pressure (mmHg)* | -0.05 | -0.18, 0.09 | 0.48 |
| Highest heart rate (bpm) | -0.15 | -0.30, -0.01 | 0.04 |
| Heart rate range (bpm)* | -0.04 | -0.23, 0.17 | 0.75 |
| Highest temperature (°C) | -1.32 | -5.24, 2.61 | 0.51 |
| SpO2 on admission (%)* | 0.46 | -0.10, 1.01 | 0.12 |
| SOFA** score^1*^  0  >=1 | Ref.  -8.36 | -15.73 -0.99 | 0.03 |
| Urine output (ml/hour) | 0.02 | -0.01, 0.05 | 0.18 |
| Creatinine (μmol/L) | 0.02 | -0.10, 0.15 | 0.75 |
| Platelets (x 10^9^/L) | -0.03 | -0.06 -0.01 | 0.01 |

*^1^SOFA score of 0 is the baseline group*

** during first 24 hours of hospital admission*

****Sequential organ failure score(19)
