# Supplementary material for: Risk factors associated with mechanical ventilation, autonomic nervous dysfunction and physical outcome in Vietnamese adults with tetanus
Source: Trop Med Health. 2021 Jun 21;49:50. doi: 10.1186/s41182-021-00336-w (PMC8215632; doi:10.1186/s41182-021-00336-w)
Supplement: Supplementary file 4 — Additional file 4. The association between independent features during ICU management and SF 36 physical function composite score at hospital discharge (n = 79). [file 41182_2021_336_MOESM4_ESM.docx]

**Additional file 4:** The association between independent features during ICU management and SF 36 physical function composite score at hospital discharge (n=79)

| **Parameter** | **Coefficient (β)** | **95% CI β** | **P-value** |
| --- | --- | --- | --- |
| Tracheostomy required | -9.68 | -13.76, -5.60 | <0.001 |
| Duration tracheostomy (days) (n=34) | -0.278 | -0.48, -0.07 | 0.01 |
| Mechanical ventilation required | -8.88 | -13.13, -4.64 | <0.001 |
| Duration mechanical ventilation (days) (n=31) | -0.23 | -0.49, 0.04 | 0.09 |
| Autonomic nervous system disturbance (ANSD) | -11.67 | -16.73, -6.61 | <0.001 |
|  |  |  |  |
| Duration diazepam required (days) (n=75) | 0.20 | -0.06, 0.47 | 0.13 |
| Duration midazolam required (days) (n=43) | -0.31 | -0.63, 0.01 | 0.05 |
| Duration Magnesium sulphate (days) (n=15) | 0.01 | -0.74, 0.77 | 0.97 |
| Duration pipecuronium (days) (n=25) | -0.35 | -0.84, 0.15 | 0.16 |
| Ventilator associated pneumonia* | -1.99 | -3.84, -0.15 | 0.04 |
| Bacteraemia* | -8.45 | -14.80, -2.11 | 0.01 |
| Urinary tract infection* | -7.51 | -13.46, -1.55 | 0.01 |
| Any healthcare associated infection | -10.63 | -14.82, -6.43 | <0.001 |
| Pressure ulcer | -6.95 | -14.39, 0.49 | 0.07 |
| Length of ICU stay (days) | -0.42 | -0.57, -0.27 | <0.001 |
| Length of hospital stay (days) | -0.35 | -0.51, -0.19 | <0.001 |

# **Infections occurring during hospitalization and deemed to be healthcare related infections.
